# Intelligent Control Techniques for the Detection of Biomedical Ear Infections

**DOI:** 10.1155/2022/9653513

**Published:** 2022-09-05

**Authors:** Mohammed J. Abdulaal, Ibrahim M. Mehedi, Abdulah Jeza Aljohani, Ahmad H. Milyani, Mohamed Mahmoud, Manish Kumar Sahu, Abdullah M. Abusorrah, Rahtul Jannat Meem

**Affiliations:** ^1^Department of Electrical and Computer Engineering (ECE), King Abdulaziz University, Jeddah, Saudi Arabia; ^2^Center of Excellence in Intelligent Engineering Systems (CEIES), King Abdulaziz University, Jeddah, Saudi Arabia; ^3^Department of Electrical and Computer Engineering, Tennessee Technological University, Cookeville, TN, USA; ^4^Department of Computer Science & Engineering, Bhabha College of Engineering, R. K. D. F. University Bhopal, Madhya Pradesh 462033, Bhopal, India; ^5^Department of Electrical and Electronic Engineering, BRAC University, Dhaka, Bangladesh

## Abstract

The capacity to carry out one's regular tasks is affected to varying degrees by hearing difficulties. Poorer understanding, slower learning, and an overall reduction in efficiency in academic endeavours are just a few of the negative impacts of hearing impairments on children's performance, which may range from mild to severe. A significant factor in determining whether or not there will be a decrease in performance is the kind and source of impairment. Research has shown that the Artificial Neural Network technique is capable of modelling both linear and nonlinear solution surfaces in a trustworthy way, as demonstrated in previous studies. To improve the precision with which hearing impairment challenges are diagnosed, a neural network backpropagation approach has been developed with the purpose of fine-tuning the diagnostic process. In particular, it highlights the vital role performed by medical informatics in supporting doctors in the identification of diseases as well as the formulation of suitable choices via the use of data management and knowledge discovery. As part of the intelligent control method, it is proposed in this research to construct a Histogram Equalization (HE)-based Adaptive Center-Weighted Median (ACWM) filter, which is then used to segment/detect the OM in tympanic membrane images using different segmentation methods in order to minimise noise and improve the image quality. A tympanic membrane dataset, which is freely accessible, was used in all experiments.

## 1. Introduction

Mid-ear and inner-ear structures in the human body are critical for sound transduction and balance, respectively. They are located in the middle and inner ears, respectively. It is first recognised by the eardrum (tympanic membrane), after which it is changed via the ossicular chain to reach the cochlea, where it is turned into a bioelectric signal and finally transferred to the brain for processing. The malleus, incus, and stapes are the three bones that make up the ossicular chain in the middle ear, which is comprised of the ossicular chain and the stapes. Ligaments and tendons link the three ossicles to the surrounding bone structures, and they are also connected to one another by two joints that attach to the rib cage. Because of this, they are capable of maintaining an intricate spatial structure while still performing the function of sound transmission. The malleus joins to the tympanic membrane, while the stapes attach to the cochlea, and both are responsible for hearing. Located around the footplate of the stapes, the stapedial annular ligament connects that bone to the oval window of the inner ear, creating a ring of fibrous tissue around it. It is positioned around the footplate of the stapes and has a rounded shape. The oval window membrane [1], which protects the opening on the cochlea that comes into contact with the stapes, is responsible for protecting the opening on the cochlea that comes into contact with the stapes during the hearing process.

Damage to the middle and inner ear caused by diseases and accidents may cause severe hearing loss in the ear that has been injured or harmed. Everywhere in the world, around 5% of the population suffers from difficulties that are caused by hearing issues. Conductive (middle ear) and sensorineural (inner ear) hearing loss are two separate types of hearing loss that may be caused by different factors (inner ear). Otitis media (also known as middle ear inflammation) is one of the most prevalent paediatric illnesses, affecting about one out of every 100 children in the United States. Beyond being a major cause of hearing loss, OM has a detrimental influence on life quality and results in a substantial increase in societal expenditures. In any given year, about 740 million people all over the world are affected by OM, with 90 percent of those affected being children under the age of 10 [2].

**Figure 1:**
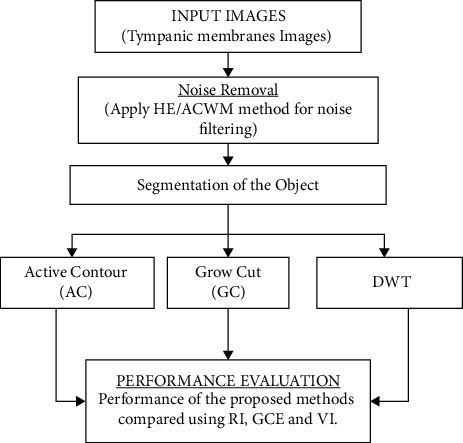
System architecture.

A conventional otoscopy device is used to check the tympanic membrane (TM) to diagnose OM in clinical practise, and a conclusion is made based on the results of the examination. Based on the changes in the TM, it is feasible to distinguish between alterations in the TM, OM, and normal tympanic membrane. The colour of the substance when used in normal conditions is grey. It has a thin and transparent appearance. Some of the most prominent symptoms of OM include redness of the eardrum and bone, along with fluid accumulation behind the tympanic membrane. OM is one of the most often reported conditions in children, and it is considered a contributing factor to the overuse and abuse of antibiotics in this population. In the first two years of life, 90 percent of children experience acute otitis for the first time, with about half of those episodes being repeated [2]. In recent years, advancements in image processing technologies have made it feasible to identify a greater variety of illnesses than previously conceivable. Among other things, while developing a computer-aided model to assist in the diagnosis of OM, it is feasible to focus on approaches such as picture preprocessing, image segmentation, feature extraction, and classification. The image segmentation system designs that were employed in this research study are shown in Figure 1, the schematic, which may be viewed in the accompanying illustration.

In light of the fact that noise is a natural property of medical imaging and that it frequently has the effect of reducing both image resolution and contrast, thereby decreasing the diagnostic rate of this imaging modality, there has been a marked increase in interest in using noise reduction techniques in a variety of medical imaging applications, which has been observed and is currently being researched. This work presents a technique for hybrid nonlinear filtering, which is divided into two sections. The first portion of the method is described as follows: first, a judgement measure that takes into account discrepancies between nearby pixel values in the input picture [3] is applied to the rank-ordered sequence to determine whether a pixel is corrupted or not in the first step. The rank-ordered sequence is then applied to determine whether or not a pixel is corrupted or not in the second step. The replacement of damaged pixels in the filtering window is carried out in the second step, utilising the centre-weighted median value of the corrupted pixels, which was determined in the first step and used to calculate the replacement of damaged pixels in the filtering window. Both the visual and experimental findings demonstrate that the proposed filter can restore images with extremely high quality while keeping image detail for photographs with varying degrees of noise density while maintaining image detail.

The proposed work involves the following section: Section 2 relates the related work and existing study, Section 3 represents the methodology of the proposed work, Section 4 represents the results and analysis of the proposed work, and Section 5 includes the conclusion and future study.

## 2. Related Works

Researchers used an otoscope with an LED light source to photograph the tympanic membrane at three different wavelengths to gather data for their study. Three different wavelengths were used: 450 nm, 530 nm, and 630 nm. Three distinct wavelengths of light were used to capture the spectroscopic images: 450 nanometers, 530 nanometers, and 630 nanometers [4–8]. It has been suggested that using such pictures in the evaluation of the tympanic membrane (TM) may make it easier to determine the boundaries of the TM and segment it with high precision [4–8].

Yasmeen and colleagues expect to release a new hybrid watermarking method in 2021 that will enhance the durability and security of digital data. The DVT and SVD [5] use multilevel procedures for both the embedding and extraction of features, resulting in a much shorter processing time overall.

A penalty term should be included in the level set function to eliminate the need for reinitialization and regularisation as a result of the use of Gaussian filtering as a reference point, according to the literature [6].

Developed using the well-established Grow Cut segmentation algorithm, the interface for applying the suggested seed point locations is based on the method's well-established Grow Cut segmentation algorithm. An extensive evaluation of the prediction ability of seed significance is carried out in this research, employing hepatic lesion input photographs, which is presented in this paper. When compared to randomly choosing the seed point position, this method results in an 8.4 percent point gain when compared to randomly selecting seed point position [7].

## 3. Noise Filtering

Processing is performed on the images to eliminate noise and other anomalies that may be found within the original image before being shown. This is done using a number of different noise filtering techniques. When the environment, the transmission channel, and/or other elements interact, noise or irregularity might arise. Following image processing, tasks such as segmentation, feature extraction, and classification are not negatively affected by the presence of noise in the picture. Picture denoising is thus essential in today's image processing systems since it contributes significantly to the overall performance of the system. Whenever you denoise a photo, you are eliminating the noise from the image to be able to view the original image once again. The presence of noise, in contrast to the ideal signal, may be caused by a broad range of reasons, including variations in detector sensitivity, the discrete nature of the radiation, environmental changes, and transmission or digitization issues, among others. It is predicted that the amount of noise created will be proportional to the number of corrupted pixels contained in the image [3, 9, 10].

### 3.1. The HE/ACWM Filter Is Used to Remove Noise

**Figure 2:**
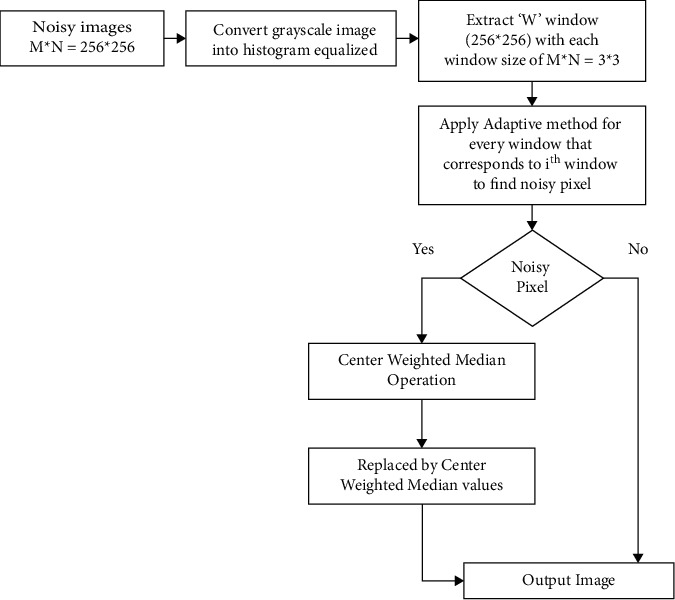
Methodology of the HE/ACWM filter.

In the past, it has been shown that median-based noise reduction techniques are more successful when dealing with fixed-valued noise, but less effective when dealing with random-valued noise. [3, 11, 12] As a consequence of this research, it has been shown that the suggested (HE/ACWM) technique consistently outperforms the competition in pictures with varied noise ratios. An instance of the block diagram of the adaptive histogram equalisation-based centr- weighted median filter technique is presented in Figure 2.

The following is a description of the four phases included in the technique proposed in this research. (1) Equalization of the histogram, (2) noise detection across the picture by setting the window size adaptively depending on the number of noise pixels inside the window, (3) equalization of the histogram and histogram equalisation, and (4) The augmentation of images center weight of each nonnoise point in the filtering window is determined in Step 3 of the filtering procedure. (5) Substitute the noise points in the image [3] with those produced by the center-weighted median filtering technique [3, 13, 14].

#### 3.1.1. Equalization of the Histogram

When determining the pathology that is being observed and making a diagnosis, high-definition medical photographs are essential. Histogram equalisation may be used to show the noise that has been hidden in a photograph after it has undergone processing. As a consequence, it is often used in the analysis of medical imaging data. It makes use of grey operations to grasp the grey mapping of pixels inside an image, which is why the histogram equalisation approach is used. The histogram is subsequently transformed into one that is uniform, smooth, and has distinct grey levels as a result of this process. For example, the image '*x*' contains the specified number of occurrences of the grey level '*I*' in the image, and the image '*y*' contains the specified number of occurrences of the grey level '*I*' in the image. The probability of a pixel of level *I* appearing in the picture is given by the following formula:

(1) $$\begin{array}{c} p_{n}\left(i\right)=\frac{n_{i}}{n},\;0\leq i<L. \end{array}$$

In regard to images, the letter ‘*L*' represents the maximum number of grey levels in the image, the letter ‘*n*_*i*_' represents the nth number of pixels and ‘*n*' represents how many pixels are in the image, and the letter *p*_*x*_ represents the image histogram for the pixel value *I* as shown in equation (2). In regard to text, the letter *p*_*x*_ represents how many pixels are in the image (*i*) as shown in equation (3). This is the CDF that matches the situation.

(2) $$\begin{array}{c} \text{CDF}{\left(i\right)}_{s}=\sum_{j=0}^{i}p_{x}\left(i\right). \end{array}$$

The CDF then will be linearized across the value range like

(3) $$\begin{array}{c} \text{CDF}{\left(i\right)}_{y}=iK. \end{array}$$

For some constant *K*, the properties of the CDF allow to perform such a transform:

(4) $$\begin{array}{c} y=\text{CDF}{\left(x\right)}_{x}. \end{array}$$

The value of the histogram of the image's input pixels is represented by the letter ‘*y'*. Finally, map the histogram values back into their original range to obtain the improved image [3].

Algorithm: the tympanic membrane serves as the input. Image with noise with a resolution of 256 × 256 pixels (histogram equalised image).

The result is a noise-free picture.

## 4. Segmentation

Segmenting images is the cornerstone of quantitative analysis in medical image processing, and it also serves as the foundation for registration, which is sometimes referred to as reconstruction in the field. The segmentation of medical pictures is essential to comprehend for medical imaging research and therapeutic applications, as well as for everyday life. It is quite difficult to do quantitative analysis on medical photos because of the rich texture and fuzzy border present in these images. Because of this, it would be required to conduct a performance review of these procedures [8, 15, 16]. This effort resulted in the development of many segmentation algorithms, which are explained further down in this section.

### 4.1. Active Contour Model (ACM)

When used in conjunction with a spatial image domain in which it has been specified, the conventional Active Contour Model, often known as the snake, is a parametric curve that can move within the picture domain. In this case, the curve is described as *p*(*s*, *t*) = (*x*(*s*, *t*)), *s*[0, 1], where it develops over time to minimise the total energy function provided by the following equation:

(5) $$\begin{array}{c} E_{\text{snake}}=\int_{0}^{1}E_{\text{int}}\left(p\left(s,t\right)\right)+E_{\text{eut}}\left(p\left(s,t\right)\right)\text{d}s, \end{array}$$

(6) $$\begin{array}{c} E_{i,j}=E_{\mathrm{int}}+E_{\text{est}}, \end{array}$$

which is repeatedly assessed to minimise the *k*_*i*_ index in the *w*_*i*_ searching window for the real discrete point using the following equation for the actual discrete point:

(7) $$\begin{array}{c} E_{\text{snake}}=\sum_{i=1}^{n}E_{i,k_{i}},\\ k_{i}=\arg\underset{j}{\min}\left(E_{i,j}\right),\;j\in W_{i}. \end{array}$$

Because the traditional ACM suffers from the limitations of initialization and the issue of local minima, shape, prior constraint has been employed to overcome these shortcomings [17]. A definition for this approach may be found in the following equation:

(8) $$\begin{array}{c} E_{T}=w_{1}E_{1}+w_{2}E_{2}+w_{3}E_{3}. \end{array}$$

*E*_*T*_ is abbreviated as *E*_*T*_. This is referred to as the shape energy *E*_2_, which is defined as the difference in form energy determined by the difference between an active contour and a shape template. It is depicted in the following way:

(9) $$\begin{array}{c} E_{2}=\int_{\Omega }{\left(H\left(\phi \right)-H\left(\varphi_{T}\left(B^{T}\right)\right)\right)}^{2}\text{d}x\text{d}y. \end{array}$$

The image domain is denoted by the symbol, as shown in the diagram. *H*(.) symbolises the Heaviside function, a denotes a signed distance function, *T* indicates a deformed template, and the transformation matrix is indicated by the translation [*tx*, *ty*]. The following notation is used to express the parameters *T*, scaling [*s*], and rotation (rot):

(10) $$\begin{array}{c} B^{T}=\left[\begin{array}{ccccccccc} 1 & 0 & t_{x} & 0 & 1 & t_{y} & 0 & 0 & 1 \end{array}\right]\times \left[\begin{array}{ccccccc} s & 0 & 00 & s & 00 & 0 & 1 \end{array}\right]\\ \times \left[\begin{array}{ccccc} s\;\sin\;\theta & \cos\;\theta & 0\;\cos\;\theta & -\sin\;\theta & 0 \end{array}\right]. \end{array}$$

In this equation, image intensity *I* and gradient operator are

(11) $$\begin{array}{c} E_{3}={\int_{\Omega }\left(\nabla H\left(\phi \right)-\nabla I\right)}^{2}\text{d}x\text{d}y. \end{array}$$

Until the difference between the previous and current segmented areas becomes stable, these three energies are evaluated iteratively until they become stable [18].

### 4.2. Grow Cut Algorithm

The cellular automata theory serves as the foundation for the Grow Cut algorithm.

#### 4.2.1. Von Neumann Neighborhood

(12) $$\begin{array}{c} N\left(p\right)=\left\{q\in Z^{n}:{\left\|p-q\right\|}_{1}≔\overset{n}{\underset{i=1}{\sum }}\left|p_{i}-q_{i}\right|=1\right\}. \end{array}$$

#### 4.2.2. Moore Neighborhood

(13) $$\begin{array}{c} N\left(p\right)=\left\{q\in Z^{n}:{\left\|p-q\right\|}_{\infty }≔\underset{i=1,n}{\max}\left|p_{i}-q_{i}\right|=1\right\}. \end{array}$$

Cell *p*'s current state, *Sp*, is represented by the triplet (*lp*, *p*, *Cp*), where *lp* represents the label of the current cell *p*, *p* represents the strength of the current cell *p*, and *Cp* represents the feature vector of the current cell *p* [19].

### 4.3. Discrete Wavelet Transform (DWT)

A mathematical approach known as deep learning (DWT) has been one of the most commonly employed in recent years for extracting information from images. In this study, the decomposition of the histogram of a picture in multiscale is accomplished by the use of the DWT concept. In the beginning, a rough value of the picture segmentation threshold is determined on a large scale, and then the scale is progressively lowered to precisely determine the segmentation threshold value. The image segmentation approach based on DWT has been shown to be successful in reducing the effects of noise. The suggested approach is based on the principle that an input picture is dissected into multiple levels of wavelet coefficients before being processed.

**Figure 3:**
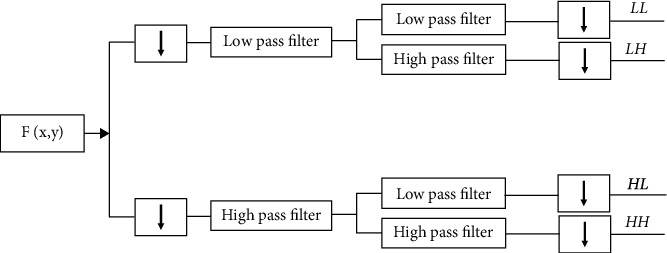
DWT processing structure.

Known as subbanding, it is the process of dividing a picture into four separate subbands that are represented by the lines in Figure 3: the LL, LH, HL, and HH bands. Changes in posture, as opposed to changes in expression, have a significant impact on the LH subband coefficients, while changes in expression have little impact on the HL subband coefficients. The coefficients of the HH subband are very sensitive to noise pollution. It is possible to divide DWT into a number of distinct varieties, each of which is characterised by the type of wavelet that is used in its calculation. The usage of a wavelet with any waveform of any shape is not practical to get the desired outcome. The usage of decomposition filters is required in order for wavelets to function successfully. It is possible to divide a vast number of wavelet types into various categories, the most fundamental of which are the Haar and Symlet wavelets, as well as the Bi-orthogonal and Reverse Bi-orthogonal wavelets. The decomposition methodology is carried out in this study using a wavelet-based Haar decomposition method, which is described in detail below [20, 21].

## 5. Results and Discussion

The purpose of this research is to compare several ways of photo segmentation. Among the segmentation algorithms taken into account in this research are the Active Contour, Grow Cut, and DWT segmentation algorithms, among others. All tactics are compared and evaluated to discover which is the most successful. It was possible to test a prototype of the suggested model using MATLAB 2018B, which operates on an Intel i7 processor with a 2 TB hard drive and 8 GB of RAM and is running on the Windows 10 operating system, by using MATLAB 2018B.

### 5.1. Dataset Description

**Figure 4:**
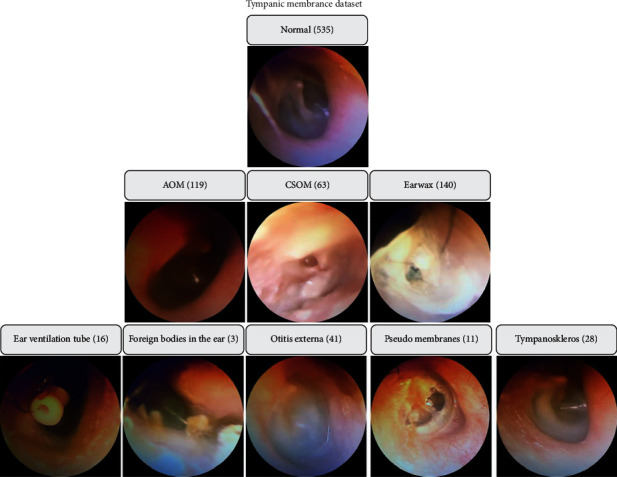
Sample TM dataset.

The dataset contains a total of 956 raw otoscope pictures that were obtained from volunteer patients who were hospitalised at the Zel Van Akdamar Hospital between October 2018 and January 2019. It was discovered that 535 normal TM samples had been collected, compared to 119 AOM samples, 63 CSOM samples, and 140 earwax samples. Aside from that, there were 41 samples from otitis externa, 16 samples from an ear ventilation tube, 3 samples from foreign bodies in the ear, and 11 samples from tympanosclerosis obtained in the other classes. To create the database, each otoscope sample was assessed by three ENT doctors. The samples belonging to distinct classes were kept in folders that were named in accordance with the OM kinds that were given. The photographs of poor quality owing to lack of light, handshake, and other factors were also removed from the database [22]. The graphic depicts a visual depiction of the TM dataset: the TM dataset as shown in Figure 4.

### 5.2. Evaluation Parameters

The performances of different methods are mentioned in the noise filtering section are compared with the various noise density ranges. The performance of the proposed noise filtering algorithm has been evaluated using various algorithms, which are shown in Table 1.

Table 1 shows the PSNR results achieved by the proposed method for *p*=30% and 50% noise density levels. However, when the noise density increases, the performance of the existing filter degrades. However, the performance of the HE/ACWM filter is better than other filters with various noise density ranges.

**Figure 5:**
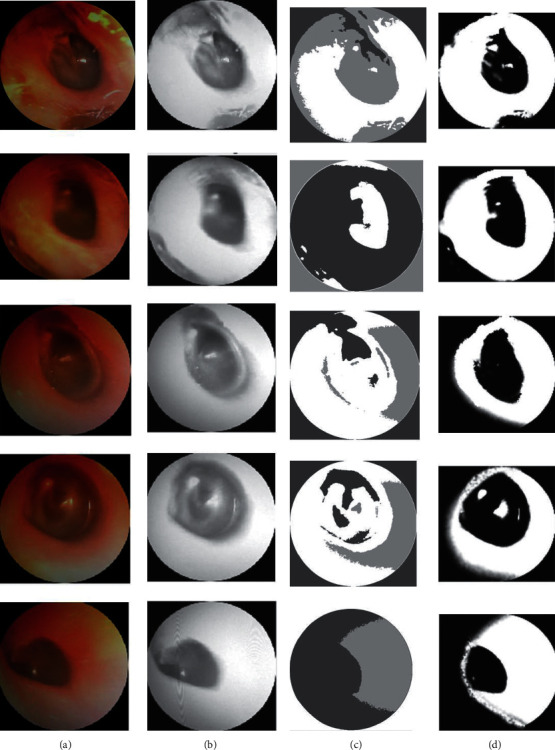
(a) Original image, (b) ACM, (c) GC, and (d) DWT.

**Figure 6:**
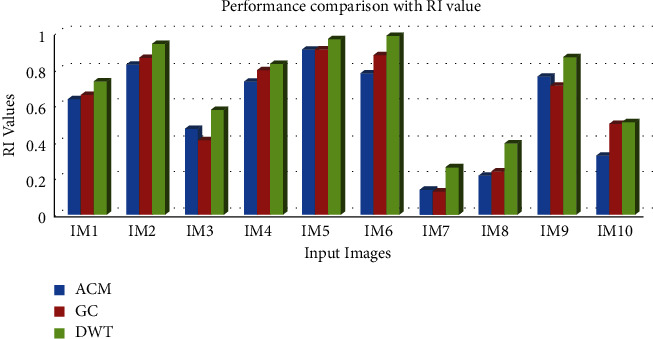
Comparative analysis of the RI value for the segmentation algorithms.

**Figure 7:**
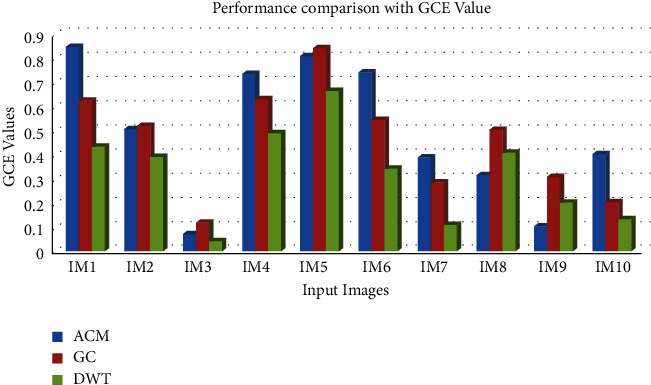
Comparative analysis of the GCE value for the segmentation algorithms.

Table 2 and Figures 5 and 6 clearly demonstrate that the suggested method has the highest RI in regard to segmenting the input images. For evaluating the performance of various input pictures, the greatest RI achieved from the DWT approach was compared with other segmentation methods, as shown in Table 3 and Figure 7.

**Figure 8:**
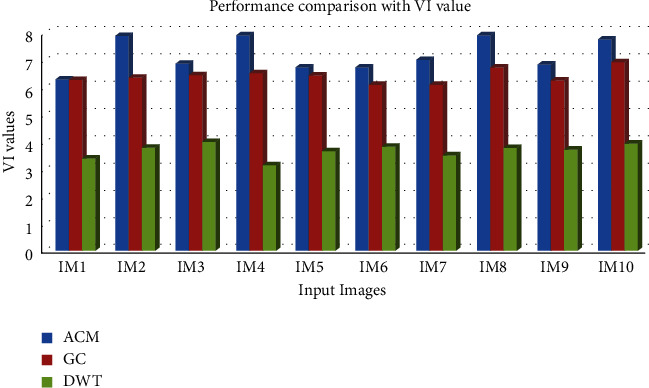
Comparative analysis of the VI value for the segmentation algorithms.

Table 4 and Figure 8 clearly demonstrate that the DWT technique has the lowest VI value when compared to other segmentation methods to analyse the performance of various input images, which is a significant finding.

## 6. Conclusion

This has the potential to result in significant and even life-threatening problems. The development of an automated computer-based image-analyzing system that might aid in establishing an accurate otitis media diagnosis anywhere is thus required. In this study, multiple segmentation approaches are used to identify otitis media from the tympanic membrane, and the results are shown. The adaptive denoising method presented in this research is a two-stage approach. In the first step, a noise detection approach that is adaptive is utilised to detect the noise. Following that, a nonlinear filter is used in conjunction with the input picture to denoise it. When compared to other segmentation approaches, the findings show that DWT-based segmentation may offer the best results when used.

## Figures and Tables

**Table 1:** PSNR-based comparison of the HE/ACWM method with other methods for denoising the input image for different density ranges.

S. no.		Weighted median filter	Adaptive median filter	HE/ACWM
IM1	30%	21.04	21.28	22.32
	40%	16.52	20.18	20.99
	50%	20.78	21.07	21.52

IM2	30%	21.57	24.71	25.72
	40%	23.26	24.48	25.13
	50%	22.11	22.97	23.09

IM3	30%	22.52	24.11	26.76
	40%	24.96	25.65	26.23
	50%	22.54	23.33	25.66

IM4	30%	21.56	22.54	23.56
	40%	25.45	26.78	27.45
	50%	22.18	22.95	24.87

IM5	30%	25.00	26.46	27.89
	40%	23.69	27.69	28.61
	50%	28.25	29.13	30.05

**Table 2:** RI comparison for different segmentation algorithms.

S. no.	ACM	GC	DWT
IM1	0.6329	0.6568	0.7325
IM2	0.8245	0.8611	0.9364
IM3	0.4732	0.4034	0.5736
IM4	0.7276	0.7918	0.8271
IM5	0.9055	0.9110	0.9664
IM6	0.7767	0.8764	0.9835
IM7	0.1354	0.1241	0.2608
IM8	0.2113	0.2374	0.3877
IM9	0.7554	0.7063	0.8654
IM10	0.3198	0.4975	0.5069

**Table 3:** GCE Comparison for different segmentation algorithms.

S. no.	ACM	GC	DWT
IM1	0.8469	0.6229	0.4334
IM2	0.5092	0.5188	0.3914
IM3	0.0695	0.1165	0.0422
IM4	0.7343	0.6311	0.4885
IM5	0.8094	0.8422	0.6655
IM6	0.7412	0.5467	0.3432
IM7	0.3885	0.2851	0.1094
IM8	0.3151	0.5041	0.4091
IM9	0.1039	0.3109	0.2009
IM10	0.4021	0.2054	0.1343

**Table 4:** Comparison of different segmentation algorithms.

S. no.	ACM	GC	DWT
IM1	6.2974	6.2844	3.4155
IM2	7.8847	6.3413	3.7859
IM3	6.8745	6.4657	3.9834
IM4	7.8934	6.5387	3.1283
IM5	6.7562	6.4391	3.6481
IM6	6.7197	6.1073	3.8264
IM7	6.9863	6.0918	3.5032
IM8	7.8756	6.7462	3.7834
IM9	6.8291	6.2472	3.7345
IM10	7.7606	6.9102	3.9365

## Data Availability

The data used to support the findings of this study are available from the corresponding author upon request.
